# mHealth Physical Activity Intervention for Individuals With Spinal Cord Injury: Planning and Development Processes

**DOI:** 10.2196/34303

**Published:** 2022-08-19

**Authors:** Sarah Victoria Clewes Lawrason, Lynda Brown-Ganzert, Lysa Campeau, Megan MacInnes, C J Wilkins, Kathleen Anne Martin Ginis

**Affiliations:** 1 School of Health and Exercise Sciences Faculty of Health and Social Development University of British Columbia Kelowna, BC Canada; 2 International Collaboration on Repair Discoveries University of British Columbia Vancouver, BC Canada; 3 Centre for Chronic Disease Prevention and Management Faculty of Medicine University of British Columbia Kelowna, BC Canada; 4 Curatio Networks Inc. Kelowna, BC Canada; 5 Community Research Partner Kelowna, BC Canada; 6 Division of Physical Medicine and Rehabilitation Department of Medicine University of British Columbia Vancouver, BC Canada

**Keywords:** exercise, stakeholder participation, spinal cord injuries, telemedicine, mobile apps, mobile phone

## Abstract

**Background:**

Interventions to support physical activity participation among individuals with spinal cord injury (SCI) are required given this population’s low levels of physical activity and extensive barriers to quality physical activity experiences.

**Objective:**

This study aimed to develop a mobile health intervention, called *SCI Step Together,* to improve the quantity and quality of physical activity among individuals with SCI who walk.

**Methods:**

Our overarching methodological framework was the Person-Based approach. This included the following 4 steps: conduct primary and secondary research (step 1); design intervention objectives and features (step 2a); conduct behavioral analysis and theory (step 2b); create a logic model (step 3); and complete the SCI Step Together program content and integrated knowledge translation (IKT; step 4), which occurred throughout development. The partnership approach was informed by the SCI IKT Guiding Principles. Three end users pilot-tested the app and participated in the interviews.

**Results:**

Step 1 identified issues to be addressed when designing intervention objectives and features (step 2a) and features were mapped onto the Behavior Change Wheel (step 2b) to determine the behavior change techniques (eg, action planning) to be included in the app. The logic model linked the mechanisms of action to self-determination theory (steps 2/3). Interviews with end users generated recommendations for the technology (eg, comparing physical activity levels with guidelines), trial (eg, emailing participants’ worksheets), and intervention content (eg, removing *graded tasks*; step 4).

**Conclusions:**

Using the SCI IKT Guiding Principles to guide partner engagement and involvement ensured that design partners had shared decision-making power in intervention development. Equal decision-making power maximizes the meaningfulness of the app for end users. Future research will include testing the acceptability, feasibility, and engagement of the program. Partners will be involved throughout the research process.

**Trial Registration:**

ClinicalTrials.gov: NCT05063617; https://clinicaltrials.gov/ct2/show/NCT05063617

## Introduction

Approximately 3 million individuals live with a spinal cord injury (SCI) worldwide, with 180,000 new cases of SCI reported each year [1]. Although most individuals who sustain a SCI use a wheelchair for mobility, a small but growing number of individuals retain the ability to walk after their injury [2]. More than half of individuals living with a SCI have an incomplete injury, meaning that motor function and ambulation recovery are possible [3]. A recent study showed that 207 out of 460 individuals with SCI sustained incomplete injuries, of which 47% ambulated some or all the time [4]. However, there is little epidemiological data on the number of individuals who ambulate for their primary mode of mobility, as there is often no follow-up after the acute injury. Individuals with SCI who walk still live with SCI-related impairments that have profound impacts on their physical, psychological, and social health. For instance, individuals with SCI who ambulate have reduced bowel function [5] and experience pain and fatigue during walking due to greater metabolic energy demands [6].

Physical activity is one behavior that can help individuals manage and overcome challenges associated with SCI-related implications. A recent review identified the following benefits of physical activity participation for individuals with SCI who walk: increased cardiovascular fitness, reduced pain and fatigue, improved cognition, and decreased depressive symptoms [7]. Although increasing the amount of physical activity is important, quality physical activity experiences are another valuable aspect of participation. Quality physical activity reflects the subjective perceptions and experiences of individuals [8]. Positive quality physical activity experiences can lead to benefits such as improved well-being, health [9], and sustained participation [10]. Although individuals with SCI who walk participate in less physical activity than those with SCI who use wheelchairs [11], there have been few attempts to help improve the quantity or quality of leisure-time physical activity for this group. Thus, interventions are required to facilitate quality physical activity behavior change among individuals with SCI who walk.

According to a meta-analysis, physical activity behavioral interventions for individuals with physical disabilities have a small to medium effect size on physical activity behavior (mean g=0.35, SE 0.07) [12]. Interestingly, most physical activity behavioral interventions for individuals with physical disabilities have been delivered in person or over the telephone, with few using digital platforms (eg, email, texting, and video) [12]. Although apps have been used to deliver other types of health interventions for individuals with SCI (eg, transitional care) [13], to the best of our knowledge, no physical activity interventions have been provided to individuals with SCI through a mobile health (mHealth) format.

mHealth interventions offer a convenient way to deliver physical activity support to individuals with SCI, especially because of the sparse and geographically distant number of individuals with SCI who walk, which makes in-person interventions nearly impossible. In addition, delivering an intervention through an app can alleviate transportation and built environment barriers to access, which are substantial for the SCI population [14]. mHealth interventions are cost-effective, available, accessible, and give users the option of flexible and convenient access to individualized programming (eg, [15]). Among able-bodied adults, a meta-analysis of smartphone-based physical activity interventions demonstrated a significant overall moderate effect size (g=0.54 for increased physical activity) [16]. Together, these data indicate the potential value of using mHealth formats (ie, smartphone apps) to deliver physical activity interventions to individuals with SCI who walk.

However, very few physical activity smartphone apps are theory based [17]. Although most existing physical activity apps use the behavior change techniques (BCTs) of goal setting, self-monitoring, and feedback, other valuable theory-based strategies are missing, such as social support, self-talk, and coping planning [18]. Indeed, the more theory-based strategies an app contains, the greater the use rates of physical activity apps [19] and the more effective they are at increasing physical activity in general [20]. In physical activity interventions for people with physical disabilities, the largest effects occurred for interventions guided by the behavior change theory [12]. Accordingly, the development of mHealth interventions to increase physical activity behavior should be explicitly informed by the behavior change theory.

Furthermore, engaging with partners throughout the development process (ie, integrated knowledge translation [IKT]) [21] is important for intervention design. For example, a physical activity counseling intervention for individuals with SCI was developed using an IKT approach and produced the largest effects reported for a behavioral intervention in this population (self-reported physical activity, Cohen *d*=1.04; peak oxygen uptake [V̇O_2_ peak], Cohen *d*=0.54) [22]. The authors attribute the success largely to the co-development process of intervention development [23]. Indeed, the authors of a recent landmark paper argued that physical activity policies, recommendations, and resources must use an IKT approach to drive greater quantity and quality of physical activity participation experiences for persons with disabilities [24].

Similarly, the Person-Based approach for intervention development provides the groundwork for developing interventions, which account for the context, perspectives, and experiences of end users through mixed methods research [25]. This approach has been used to guide the development of many digital health interventions as a primary goal of this method is to increase the feasibility, acceptability, and engagement of interventions [26]. Notably, the Person-Based approach is used complementarily with theory- and evidence-based approaches [15,26]. However, a limitation is that it does not provide guidance on when and how to include partners in the intervention development process. Greater clarification on how to implement partner engagement using IKT in combination with Person-, theory-, and evidence-based approaches is needed.

Recently, 8 IKT guiding principles for SCI research (refer to Gainforth et al [27] for more details) were developed to address the gap in guidance on partner engagement. These principles help researchers to engage meaningfully in partnered research that is relevant and usable [27]. Using these principles to guide IKT has the potential to reduce experiences of tokenism, increase the coproduction of knowledge, and create meaningful SCI research partnerships [27].

The purpose of this paper is to describe the 4-step intervention planning and development processes of an mHealth intervention to increase the quantity and quality of physical activity participation among individuals with SCI who walk. This paper provides an illustration of how the Person-Based approach can be used alongside IKT, theory, and evidence to plan and develop an mHealth intervention. Of note, this paper reports on the processes of steps 2 to 4, whereas step 1 is summarized because these details are reported elsewhere [7,28,29].

## Methods

### Context

Our intervention, SCI Step Together, was developed as a program within an existing app called *Stronger Together*. The Stronger Together app was created through partnership with Curatio Networks Inc and the University of British Columbia to create communities of peer support for self-management of various chronic conditions (eg, patients who have undergone knee replacement surgery, adults with physical disabilities, and COVID-19 long-haulers). The platform takes a differentiated approach to behavioral change by embedding social connections into health literacy and management interventions. The platform is privacy and regulatory compliant to meet the requirements of life sciences policy and protocols. The Stronger Together platform has already undergone usability and acceptability testing privately through Curatio Networks Inc. Overall, the app provides users access to educational modules, health tracking, peer support, and individual behavioral support from a live community coach.

**Figure 1:**
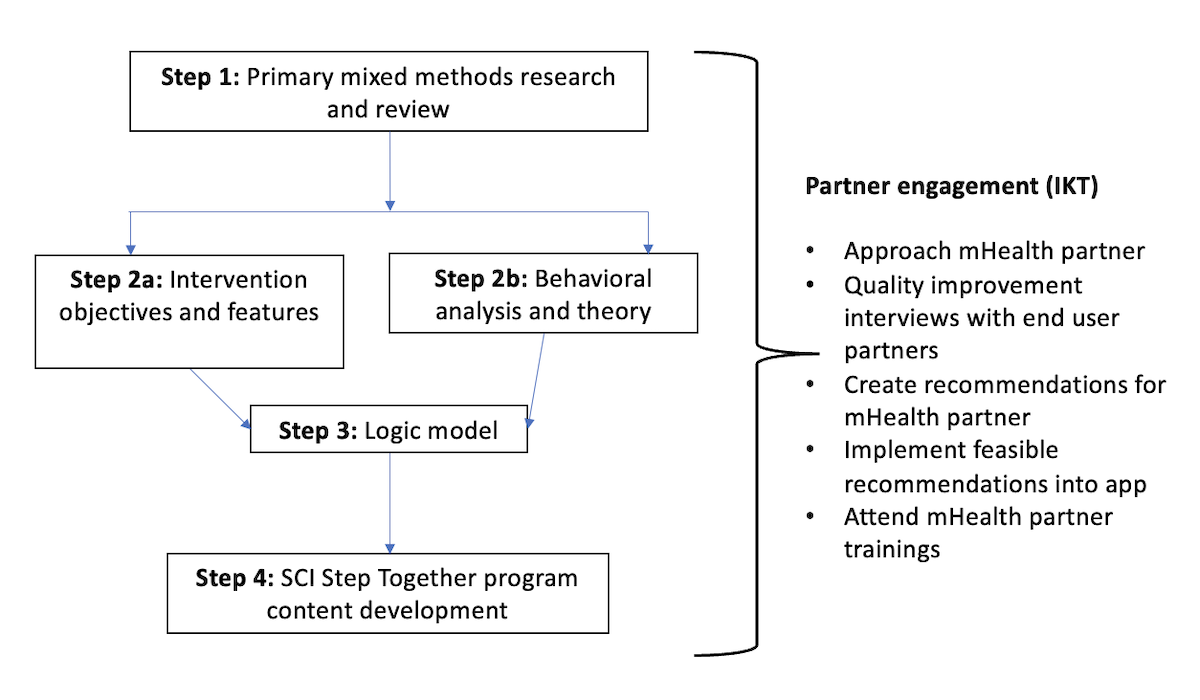
Steps in intervention planning and development process for the SCI Step Together program. Adapted from Band et al [15]. IKT: integrated knowledge translation; mHealth: mobile health; SCI: spinal cord injury.

In the following sections, we describe the methods for planning, adapting, and developing the SCI Step Together intervention. Adapted from the study by Band et al [15], Figure 1 shows each step in the intervention planning and development process, including partner engagement activities to demonstrate the IKT approach. Importantly, evidence from primary studies and systematic reviews was incorporated throughout the intervention development process, with the intervention development steps being updated and carried out iteratively. This included four steps: (1) conduct primary mixed methods research and review, (2a) design intervention objectives and features, (2b) conduct behavioral analysis and theory, (3) create a logic model, and (4) complete the SCI Step Together program content development. In addition, partner engagement occurred throughout the intervention planning and development process and will be described in step 4.

### Step 1: Primary Mixed Methods Research and Review

Step 1 involved conducting three studies: a scoping review, a quantitative cross-sectional study, and a qualitative study. The purpose of step 1 was to gain an understanding of the background and context of physical activity participation for people living with SCI who walk. The goal was to identify factors to target in the intervention along with recognizing the context of lived experience for end users. The methods for step 1 are not novel and are only described briefly. Importantly, IKT was incorporated into all 3 studies and is described in detail elsewhere (along with the methods and results) [7,28,29].

#### Scoping Review

The purpose of the scoping review was to understand the amounts, types, correlates, and outcomes of physical activity participation for individuals with SCI who ambulate as their primary mode of mobility. IKT was used by consulting individuals with SCI who walk and a provincial SCI organization to determine research questions and search strategies. Using a published method for scoping reviews [30], a systematic search was conducted among academic (MEDLINE, PsycINFO, Embase, CINAHL, Web of Science, and Sport Discus) and gray literature (Open Access Theses and Dissertations and ProQuest Dissertations and Theses) databases, yielding 3257 articles. After a 2-phase screening process by 2 independent coders, 17 articles were selected for inclusion, and the data were charted and summarized, and correlates were coded using the Capability, Opportunity, Motivation-Behavior (COM-B) model [31]. The COM-B model identifies the source of the behavior, which is the first step in the Behavior Change Wheel (BCW; see below in Step 2b for more detail) [31], a theoretically aligned approach to developing interventions.

#### Primary Mixed Methods Research

Two studies were conducted with a sample of adults with SCI who self-reported that they walked for daily mobility more often than not (ethics board approval time: 52 days). IKT was used by partnering with individuals with SCI, a behavior change theory expert, and a provincial SCI organization to determine measure selection and adaptation, recruitment, and data analysis. The first study (n=43) used a cross-sectional design and required 1.5 hours of time from participants. This study used the Theoretical Domains Framework [32] to identify behavioral change factors related to physical activity participation among ambulators with SCI. The Theoretical Domains Framework is embedded in the COM-B model. The Theoretical Domains Framework domains are used to assess capability-, opportunity-, and motivation-related barriers and facilitators to engage in a behavior [33]. A modified version of the validated Determinants of Physical Activity Questionnaire [34] was used to assess behavioral change factors. We also measured the duration, type, and intensity of physical activity performed during the previous week using the Physical Activity Recall Assessment for individuals with SCI [35,36]. This measure has demonstrated adequate test-retest reliability and evidence of criterion and construct validity [35-37].

The second study used a qualitative approach to explore the conditions and elements involved in quality physical activity experiences. Semistructured interviews were conducted with 22 participants in the cross-sectional study. The interviews lasted for approximately 60 minutes and were transcribed verbatim. Using a philosophically pragmatic approach [38], the data were reflexively thematically analyzed [39] with the goal of informing the SCI Step Together intervention. The data were first inductively coded by 2 independent coders, with feedback from critical friends. Then, the data were deductively coded using the Quality Parasport Participation Framework [40] to identify conditions and the Quality Participation Framework [41] to identify elements of quality participation.

### Step 2a: Intervention Objectives and Features

An essential component of the Person-Based Approach [25], the purpose of this step was to develop key intervention objectives and design needs, along with associated intervention features that address these objectives.

First, we stated the objectives of the intervention with respect to behaviors and outcomes and described the relevant aspects of users and their context, as informed by step 1 [25]. In addition, we identified the key behavioral issues, needs, and challenges that the intervention must address. Second, we formulated the intervention design objectives according to the evidence generated in step 1. Third, we generated the design features intended to achieve each objective based on the intervention planning, evidence from step 1, behavioral analysis from step 2b, and logic model from step 3. These objectives and features have been refined throughout the intervention planning and development process based on partner engagement and evidence (eg, inclusion of theory).

### Step 2b: Behavioral Analysis and Theory

The purpose of step 2b was to use behavior change theory to identify BCTs to be included in the intervention content as well as to choose a specific theory to inform intervention content.

#### Behavioral Analysis

The behavioral analysis was conducted using BCW [31], which is a theoretical framework that guides researchers to identify the source of behavior (ie, capabilities, opportunities, and motivations) and then links these to the selection of intervention functions, policy categories, and BCTs. The results from the quantitative study examining the Theoretical Domains Framework behavior change factors (step 1) were used to inform the behavioral analysis. First, the Theoretical Domains Framework domains (predictors of, and barriers to, physical activity) were linked to intervention functions. The most common and feasible intervention functions were identified, and these were linked to common and feasible policy categories. The intervention functions and Theoretical Domains Framework domains were then linked to BCTs, noting the most frequently used BCTs [42]. Finally, 2 reviews of the effectiveness of BCTs in physical activity and self-management interventions among individuals with SCI and physical disabilities generally were consulted [12,43]. These reviews allowed us to decide which BCTs should be included or excluded based on their effectiveness in similar interventions.

#### Theory

In addition to conducting a behavioral analysis and identifying BCTs for inclusion in the intervention, we selected a theory to inform the intervention. Using theory helps to outline the critical assumptions that underlie the intervention protocol, the primary constructs that are effective, and the causal processes tested by mediators [44]. The first author (SL) reviewed theories on changes in physical activity behavior [45]. In addition, discussions occurred among the first and senior authors (experts in exercise psychology) regarding theory fit and choice. We considered the evidence identified in primary mixed methods studies (step 1; ie, Theoretical Domains Framework domains to target and context from qualitative interviews) in addition to the intervention objectives identified in step 2a.

### Step 3: Logic Model

The purpose of developing the logic model was to clarify the hypothesized causal relationships and mechanisms of action that mediate intervention outcomes [46]. The logic model was created in accordance with the Medical Research Council evaluation guidance [46] and process evaluation methods [47]. The logic model included the intervention inputs, processes or actions, outputs, and outcomes. Having clear intervention objectives and features enabled us to understand the problems that needed to be addressed and to select appropriate intervention components. The BCTs identified in the behavioral analysis in step 2 were included as intervention inputs in the logic model, whereas the theory selected in step 2 informed all aspects of the logic model’s causal assumptions. The logic model underwent several iterations as the development process continued to include feedback from all partners involved.

### Step 4: SCI Step Together Program Content Development and IKT

The partners involved in the development of the SCI Step Together program included 2 SCI physical activity behavior change scientists (SL and KMG); an external project manager at Curatio, Inc, the company that hosts the technology platform (LC); the CEO of Curatio, Inc (LBG); and potential end users of the program (ie, ambulators with SCI; JS, MM, and CJ). The partners included both women (n=5) and men (n=2) and were both over (n=2) and under (n=5) the age of 50 years.

The SCI Step Together program is hosted on Curatio’s Stronger Together platform, which was developed and tested among other users with physical disabilities. Six months after the launch of Stronger Together (January 2021), the senior author (KMG) approached Curatio, Inc (LBG), and the 2 partners agreed to create a community for ambulators with SCI using an evidence- and theory-based approach.

One of the original (January 2021) Stronger Together communities is for adults with physical disabilities who want to increase their physical activity. The first author (SL) reviewed the content modules for this community. SL then revised the modules for SCI Step Together, based on the information collected from steps 1 to 3 and content from other physical activity interventions for individuals with SCI, including the ProActive SCI Toolkit [23], Active Homes [48], the Blueprint for Quality Participation [49], and the SCI Physical Activity Guidelines [50].

The first author (SL) and senior author (KMG) wanted to engage end users to receive feedback on the preliminary intervention content and delivery. Accordingly, the first author (SL) developed an interview guide to conduct partnered quality improvement of the program content. Three potential end users (ambulators with SCI) were interviewed, all with different levels of physical activity participation. The purpose of the interviews was to produce recommendations for the format and general content of this intervention. Before the interviews, the end users were asked to review the original Stronger Together program (physical activity for people with physical disabilities) to understand how the app works and its various components. In addition, end users were sent lay summaries of the (step 1) studies informing intervention development. Finally, we sent the end users the 8 IKT guiding principles for conducting quality and ethical SCI research (Multimedia Appendix 1) [27]. These principles are intended to be used by all partners early and throughout the research process [27]. The IKT guiding principles were discussed with the end users at the beginning of the interview to ensure that all partners were aligned with how the intervention development process would occur. The interviewer (SL) took notes throughout the interview to record recommendations.

Following the interviews, the first author (SL) met the project manager from Curatio. During the meeting, they discussed the project and timelines, and they reviewed the IKT guiding principles. The first author (SL) brought forward technology-related recommendations of the end users on how the original Stronger Together program could be adapted and improved for SCI Step Together. Recommendations were sent to the project manager and software developers who returned a list of what could and could not be changed. Ultimately, the recommendations from end users were incorporated as best as possible for both content and delivery, with the decisions reported in the Results section. These decisions were then communicated back to the SCI end users. Importantly, both the technology and SCI end user partners have been included as authors of this manuscript.

In addition, Curatio invited the first author (SL) to attend trainings on how to engage with participants, technology information, and how to record and manage time in addition to privacy concerns.

### Ethics Approval

Ethical approval was not applied for to develop program content as it was completed for quality improvement. Under Article 2.5 of the Tri Council Policy Statement, quality assurance or quality improvement activities are not subject to institutional ethical review.

## Results

### Step 1: Primary Mixed Methods Research and Review

The results of the step 1 studies have been published elsewhere [7,28,29]. Key issues identified from the primary mixed methods research and scoping review are summarized in Table 1. The implemented intervention features addressing each issue are also reported in Table 1.

**Table 1:** Key issues from primary mixed methods research and scoping review and associated intervention features.

Issue identified by research	Intervention features addressing the issue
Ambulators with SCI^a^ participate in low levels of leisure-time physical activity, and no interventions exist for this group [7].	Intervention should be developed to improve physical activity participation.
Exercise intervention studies lacking measurement of psychosocial outcomes [7].	Intervention must address and measure psychosocial constructs related to physical activity participation.
Correlates related to physical activity include physical and psychological capability (eg, pain and lack of knowledge), environmental and social opportunity (eg, time and underestimated disability), and reflective and automatic motivation (eg, intentions and boredom) [7] Barriers to physical activity include lack of knowledge, weak beliefs about capabilities, lack of coping planning, and high goal conflict [28]. Coping planning, action planning, goal conflict, and skills significantly predict physical activity [28].	Intervention must target the following constructs through educational modules, behavioral support, and peer support: Physical activity guidelines and benefits (knowledge) Self-monitoring and goal setting (goal conflict) Action planning Coping planning Confidence (beliefs about capabilities) Skills
A total of 35 types of physical activity recorded and organized into 10 higher-order categories (eg, walking, resistance training, and rock climbing) [28].	Intervention content must include educational modules that refer to these types of physical activity when examples are used. A list of these types of physical activity as ideas for participants should also be included.
Ambulators with SCI have physical activity experiences, which are shaped by feelings of ableism, feeling sidelined, and the effects of their SCI [29].	Behavioral support in the intervention must be provided by a person who understands this context for physical activity participation.
Conditions and elements of quality physical activity experiences map onto the Quality Participation Framework [41] and Quality Parasport Participation Framework [29,40].	Conditions and elements of quality physical activity experiences must be included as a separate module for intervention. Quality of physical activity experiences must be referred to throughout the intervention.
Ambulators with SCI lack sense of community, especially in physical activity settings [29].	Peer support must be included and prioritized in intervention delivery.

^a^SCI: spinal cord injury.

### Step 2a: Intervention Objectives and Features

The objective of the SCI Step Together program, in terms of outcomes, is to increase the quantity and quality of leisure-time physical activity participation among persons with SCI who ambulate. The implemented intervention objectives and features based on our understanding of the key behavioral issues can be found in Table 2.

**Table 2:** Intervention objectives and features for the SCI^a^ Step Together program.

Intervention design objectives	Key features
To increase the quantity of physical activity among individuals with SCI who ambulate	Digital intervention to build autonomous motivation through teaching self-regulation skills (eg, action and coping planning) and increasing autonomy, competence, and relatedness Provide educational information on physical activity and self-regulatory behaviors Behavioral coach provides support to individuals through BCTs^b^ such as feedback on behavior and verbal persuasion.
To enhance the quality of physical activity among individuals with SCI who ambulate	Increase intrinsic motivation to participate in activities that align with their desires, needs, and lifestyles through building autonomy, competence, and relatedness Provide resources and support to individuals with SCI who ambulate through behavioral coaching and peer support to offer additional options that may be relevant for their context Provide education on quality participation elements and factors to increase opportunities for experiencing quality in physical activity
To build community among individuals with SCI who ambulate	Allow individuals to communicate in the app to offer support to each other and gain an awareness of others in their situation

^a^SCI: spinal cord injury.

^b^BCT: behavior change technique.

### Step 2b: Behavioral Analysis and Theory

The theory chosen to inform the SCI Step Together program was the self-determination theory (refer to the study by Ryan and Deci [51] for a review), which is a theory of human motivation and personality that posits that three basic psychological needs must be met for a person to sustain a behavior (eg, physical activity): autonomy, competence, and relatedness [51]. Self-determined or autonomous motivation occurs when these 3 needs are met and individuals are intrinsically motivated [51]. This theory was chosen to inform the intervention content because the self-determination theory aligns well with not only increasing the quantity but also the quality of physical activity participation. We hypothesized that there is a positive correlation between intrinsic motivation for physical activity and quality physical activity participation.

Importantly, all barriers and facilitators (ie, Theoretical Domains Framework domains) identified in step 1 can be targeted through self-determination theory constructs. For example, beliefs about capabilities and skills are two domains encompassed in the basic psychological need of competence. Furthermore, a recent review of health interventions maps BCTs onto basic psychological needs [52], making it possible to further align the work completed in step 1 with self-determination theory. Basic psychological needs are incorporated as part of the intervention objectives and features in step 2b. In addition, the included BCTs and the associated Theoretical Domains Framework domains and intervention functions can be found in Multimedia Appendix 2 [31,42,53]. The BCTs, their support for inclusion (or exclusion), and the associated basic psychological needs can be found in Multimedia Appendix 3 [12,43,52,54]. Multimedia Appendix 4 provides the BCTs, related intervention components, and rationale for how they are included in the intervention.

### Step 3: Logic Model

**Figure 2:**
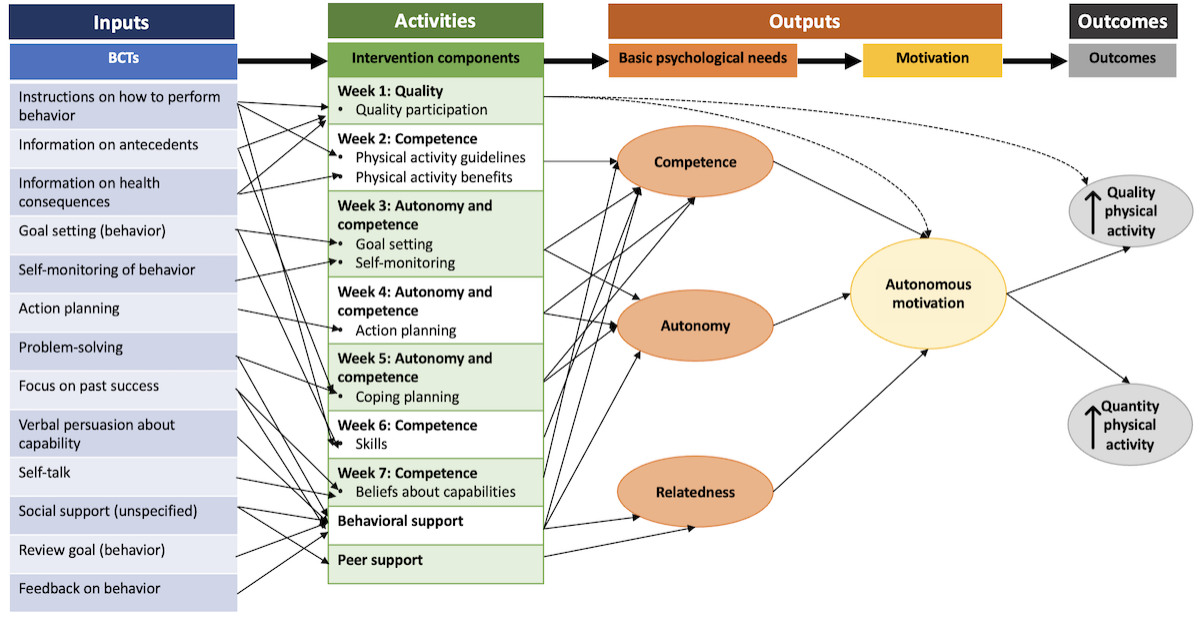
The logic model for the spinal cord injury (SCI) Step Together program. BCT: behavior change technique.

The logic model for the intervention is illustrated in Figure 2. The intervention inputs are the BCTs, the processes or actions are the intervention components, the outputs are the fulfillment of the basic psychological needs and motivation, and the outcomes are the quality and quantity of physical activity participation. The logic model presents the causal assumptions and theory of change within the intervention based on self-determination theory constructs and includes BCTs and intervention components identified from steps 1 to 2.

### Step 4: SCI Step Together Program Content Development and IKT

No training sessions were provided to end users, as they felt comfortable navigating the app on their own. End users spent 1 to 2 hours using the Stronger Together program to become familiar with app use and engagement required. The initial interview with end users resulted in several recommendations for the final SCI Step Together program (Table 3). In general, end users (pilot participants) enjoyed the app, found it easy to use, and experienced no technical issues. They appreciated how the community coaches are real, live people who can provide individualized feedback rather than standardized technological support. End users also liked the inclusion of peer support, noting its importance for participating in physical activity. All end users agreed that asking participants to engage with the program once per week was preferred, rather than daily or biweekly. They thought that the images used in the app were appropriate and the general content included in the educational modules was well done.

**Table 3:** End user recommendations and changes for the SCI^a^ Step Together program.

Partner			Recommendation		Curatio feedback		Implemented (yes or no)		How
**Technology**									
	SL	Allow worksheets to be completed interactively within the app rather than as a PDF image		Not possible		No		—^b^	
	SL	Track only exercise (no other behaviors such as smoking)		Not possible		No		—	
	SL	Include physical activity planners or goal setting in the app instead of tracking medications		Possible to remove medication tracking but not possible to include planner or goal setting		Yes		Removed medication tracking. Participants can set and track physical activity goals in the “Notes” section of the app under “My Info.”	
	MM	Add the type of physical activity when tracking exercise		Unable to do		No		—	
	MM	See how your weekly physical activity compares with the guidelines every week		Unable to do within the app platform, but coach can compare		Yes		Coach will note comparisons with SCI physical activity guidelines when providing feedback on their behavior.	
	JS and CJ	Allow exercise tracking in the SCI Step Together app to sync with your smartphone activity or other fitness apps (eg, Strava)		Not possible from software standpoint		No		—	
	JS	Create a calendar to plan physical activity that syncs with iCal or Google Calendar		Not possible from software standpoint		No		—	
	SL	Change intensity from “Low” and “High” to “Mild,” “Moderate,” and “Vigorous” to align with the SCI physical activity guidelines		Not possible		No		—	
	SL	Allow participants to self-select minutes of exercise rather than choosing “0-10,” “11-20” etc		Not possible		No		—	
**Trial**									
	MM	Email participants the worksheets in addition to having them accessible in the app		Possible to do by researchers		Yes		Researcher will ask participants if they would like their weekly worksheet emailed.	
	MM and CJ	Give people choice in the modules they complete		Possible from research standpoint		Yes		Participants will be encouraged to complete all the modules, but they do not have to complete them all.	
	MM, JS, and CJ	Allow people to choose how often they would like to be notified by the community coach		Possible		Yes		Community coach will ask participants how often they would like to be reminded of completing the modules	
**Intervention content**									
	MM	Provide examples of strategies to be physically active at home		Possible to include in educational content (researchers)		Yes		Educational content uses examples throughout based on physical activity at home	
	MM, JS, and CJ	Include supportive, motivational, and individualized messages from the community coach		Possible		Yes		Community coach will use autonomy-supportive messages that are individualized to each user’s profile.	
	JS and CJ	Remove *graded tasks* BCT^c^—can be overwhelming for individuals new to physical activity		Possible		Yes		*Graded tasks* BCT removed from educational modules and community coach content	

^a^SCI: spinal cord injury.

^b^Not available.

^c^BCT: behavior change technique.

As seen in Table 3, Curatio, Inc could not implement several technology-related recommendations. This is because the platform is already commercially available and hosts several communities in addition to SCI Step Together. Changing this technology would require substantial time, money, and human resources, which are not feasible for the academic or technology partners. As such, these recommendations will be considered in future iterations of Stronger Together communities.

Ultimately, SCI Step Together was designed as an 8-week program that consists of educational modules, peer support, and in-app behavioral support with a live community coach. For SCI Step Together, the first author serves as the community coach. The coach checks in weekly with participants using an autonomy-supportive approach to align with the self-determination theory [51], delivers appropriate BCTs (see the logic model in Figure 2), and provides support with the educational modules and app technology as needed. The educational modules incorporate BCTs and include links to local resources and worksheets so that participants can put their work into practice. Participants can provide and receive peer support by talking to each other in the app.

## Discussion

### Principal Findings

This paper provides the stepwise methodology used to develop the SCI Step Together intervention using a Person-, evidence-, and theory-based approach. Importantly, the approach transparently describes how various partners were involved throughout the intervention planning and development process using IKT. The intervention was systematically created with evidence generated from primary mixed methods and review research (step 1), which informed the behavioral analysis and theoretical modeling (step 2) to produce a logic model (step 3). Steps 1 to 3 along with partnership discussions using IKT helped to develop and optimize the Stronger Together program content and delivery (step 4). Overall, this intervention was developed in partnership to provide more meaningful, better-informed, and more relevant resources and support for physical activity participation to individuals with SCI who walk.

This study offers important contributions to the mHealth literature by outlining how programs can be planned and developed in partnership with partners, while using evidence, a Person-Based approach, and theoretical modeling. Although a growing number of physical activity interventions have been developed for individuals with SCI, this is the first intervention delivered in an mHealth format. Furthermore, considering that more than 50% of mHealth interventions do not incorporate theory or BCTs [18,55], this intervention development paper provides guidance on how to incorporate these important elements. The SCI Step Together program not only includes BCTs according to a behavioral analysis using the BCW [31] but is also directly guided by the self-determination theory [51] to inform causal assumptions. BCTs were also included and excluded based on previous reviews of effectiveness [12,43], thus adding confidence in the potential utility of the intervention. BCTs can be delivered and enacted appropriately using mobile technologies [56]. For example, the BCT of self-monitoring may be easier to implement through mHealth formats because users’ phones are ready and accessible immediately after exercising [57]. As such, self-reported physical activity measures may have increased precision [58], allowing participants to set more appropriate goals and coaches to provide better feedback and physical activity behavioral support [59].

This study also contributes to the mHealth literature by outlining an mHealth intervention development method that may be useful in promoting app engagement. Engagement with apps can defined as “(1) the extent (eg, amount, frequency, duration, and depth) of use and (2) a subjective experience characterized by attention, interest, and affect” [60]. Engagement with mHealth programs is typically poor, which leads to insufficient behavior change (eg, [61]). Factors that increase engagement in mHealth interventions include using BCTs (eg, self-monitoring and action planning) and providing health care practitioner support [60,61]. By conducting a behavioral analysis and working with a technology partner who endorses social support from a health coach, the SCI Step Together program is more likely to support participant engagement. Other factors that influence app engagement include “safety netting” (ie, having the ability to re-engage with the app after disengaging) and tailored content [60,61]. Using IKT allowed us to work with end users to create tailored physical activity content that could be individualized in the app through the health coach. Furthermore, working in partnership with Curatio Inc means that the SCI Step Together program can be sustained after the intervention is tested, providing a “safety net” by allowing individuals to disengage and re-engage with the program whenever they please. As such, by including behavioral analysis and IKT in the intervention development process, participants are more likely to engage positively with the program.

In addition, this is one of the first mHealth interventions to explicitly report IKT activities conducted during intervention development. Similarly, this is the first study to use the IKT guiding principles for SCI research [27] to guide our research partnership. Partners were involved throughout steps 1 to 4 to provide feedback on program content and delivery. The involvement of partners best predicts the translation of research into practice [27,62], and it is becoming increasingly important in physical activity intervention development practices. This study outlines how IKT can add to the Person-Based approach by using principles to guide partnership [27], ensuring partners have shared decision-making power in intervention development, describing who and when partners were involved, and involving partners early and throughout the research process.

### Future Directions

This paper describes the formative work of planning and developing an mHealth intervention to increase the quantity and quality of physical activity among persons with SCI who ambulate. As such, the next step, according to the Medical Research Council guidance on developing complex interventions [63], is to pilot-test the acceptability, feasibility, and engagement of the SCI Step Together program among a sample of users. This pilot study is currently underway, and the trial is registered at ClinicalTrials.gov (NCT05063617) [64]. The goal of the pilot study was to gain an understanding of how individuals used the program, whether they were satisfied with the components, and any areas for improvement before running a larger trial. In addition, we hope to estimate the effects of the intervention on key outcomes to determine a sample size for a future trial. If these feasibility and subsequent effectiveness trials are successful, the next step is implementation, whereby the program will be disseminated among the larger community and long-term outcomes will be monitored [63]. Finally, future research is needed to understand if and how BCTs are enacted by participants in an mHealth format and whether BCT enactment relates to intervention outcomes [56].

Importantly, as we move forward, the Person-Based [25] and IKT approaches [21] will continue to be used to ensure that partners are involved throughout the intervention evaluation process. For example, community and provincial SCI organizations will be involved in participant recruitment for the pilot trial and will be involved in the long-term implementation of the program. In addition, the program will be iteratively updated as mixed methods research continues to be used for program evaluation [25].

### Limitations

Some limitations of the intervention planning and development processes should be recognized. First, the studies in step 1 were conducted before the COVID-19 pandemic, and the intervention was released in July 2021. As such, some factors related to the quantity and quality of physical activity participation may differ due to social distancing, facility closures, and other public health measures. We have included several examples of at-home physical activity strategies in the SCI Step Together program, considering pandemic-related restrictions. Second, a relatively small sample of participants was used in step 1 and for partner interviews in step 4, and their preferences for programming may be limited to this sample. However, we included evidence from larger systematic reviews to inform theoretical content (eg, [43]) and build from previous interventions for individuals with SCI (eg, [23]); thus, we expect the program to be beneficial for a broader sample of individuals with SCI who walk. Third, no studies have been conducted to understand whether and how self-determination theory and quality participation are related. We do not know whether these hypothetical causal assumptions will work as expected, and larger-scale studies are required to test the relationship between self-determination theory and quality participation. Fourth, we acknowledge that many of the cocreated ideas and recommendations developed using the IKT process were not incorporated into the intervention because of the limited capacity of the technology and thus lacked feasibility for implementation. Finally, our partners were limited in racial and ethnic representation, and future partners should include a more diverse sample of individuals with SCI who walk.

### Conclusions

This is the first physical activity mHealth intervention for persons with SCI and the first physical activity intervention designed specifically for people with SCI who walk. This study provides an innovative methodology for intervention planning and development that integrates Person-Based approach, evidence, theory, and IKT. The SCI Step Together program was developed in partnership with partners to optimize intervention content and delivery. Accordingly, it is anticipated that the SCI Step Together program will be acceptable, feasible, and have appropriate engagement when pilot testing is underway.

## Appendix

Multimedia Appendix 1 Integrated knowledge translation guiding principles [27].

Multimedia Appendix 2 How behavior change techniques target Theoretical Domains Framework domains and intervention functions.

Multimedia Appendix 3 How behavior change techniques may target basic psychological needs and evidence for use in interventions.

Multimedia Appendix 4 Outlining how behavior change techniques are incorporated into the intervention content.

## Abbreviations

BCT: behavior change technique
BCW: behavior change wheel
COM-B: Capability, Opportunity, Motivation-Behavior
IKT: integrated knowledge translation
mHealth: mobile health
SCI: spinal cord injury
