# Supplementary material for: mHealth Physical Activity Intervention for Individuals With Spinal Cord Injury: Planning and Development Processes
Source: JMIR Form Res. 2022 Aug 19;6(8):e34303. doi: 10.2196/34303 (PMC9440410; doi:10.2196/34303)
Supplement: Multimedia Appendix 1 [file formative_v6i8e34303_app1.docx]

**Appendix 1: IKT Guiding Principles (Gainforth et al., 2021)**

Below are the eight IKT guiding principles for conducting SCI research in partnership. Please see <https://ikt.ok.ubc.ca/> for more information.

1. Partners develop and maintain relationships based on trust, respect, dignity, and transparency.
2. Partners share in decision-making.
3. Partners foster open, honest, and responsive communication.
4. Partners recognize, value, and share their diverse expertise and knowledge.
5. Partners are flexible and receptive in tailoring the research approach to match the aims and context of the project.
6. Partners can meaningfully benefit by participating in the partnership.
7. Partners address ethical considerations.
8. Partners respect the practical considerations and financial constraints of all partners.
