# Supplementary material for: mHealth Physical Activity Intervention for Individuals With Spinal Cord Injury: Planning and Development Processes
Source: JMIR Form Res. 2022 Aug 19;6(8):e34303. doi: 10.2196/34303 (PMC9440410; doi:10.2196/34303)
Supplement: Multimedia Appendix 2 [file formative_v6i8e34303_app2.docx]

**Appendix 2:** How BCTs target Theoretical Domains Framework domains and intervention functions

| **BCT** | **Theoretical Domains Framework Target** | **Intervention Function** |
| --- | --- | --- |
| Instructions on how to perform behaviour | Skills | **Training** |
| Information on antecedents | Knowledge | Education |
| Information on health consequences | Knowledge | **Education** |
| Goal setting (behaviour) | Goals | **Enablement** |
| Self-monitoring of behaviour |  | **Enablement, Training, Education** |
| Action planning | Goals | **Enablement** |
| Problem solving | Behavioural Regulation | **Enablement** |
| Focus on past success | Beliefs about Capabilities, Optimism | Enablement |
| Verbal persuasion about capability | Beliefs about Capabilities | Enablement |
| Self-talk | Beliefs about Capabilities | Training, Enablement |
| Social support (unspecified) | Social Influences | **Enablement** |
| Review goal (behaviour) | Goals | **Enablement** |
| Feedback on behaviour | Knowledge | **Training, Education** |

Note: Adapted from [1], [2]. Bold = most frequently used in behaviour change interventions according to [3]; Unbolded = less frequently used

**References**

[1] S. Michie, M. M. van Stralen, and R. West, “The behaviour change wheel: A new method for characterising and designing behaviour change interventions,” *Implementation Science*, vol. 6, no. 1, p. 42, 2011, doi: 10.1186/1748-5908-6-42.

[2] S. Michie *et al.*, “The Behavior Change Technique Taxonomy (v1) of 93 Hierarchically Clustered Techniques: Building an International Consensus for the Reporting of Behavior Change Interventions,” *Annals of Behavioral Medicine*, vol. 46, no. 1, pp. 81–95, Aug. 2013, doi: 10.1007/s12160-013-9486-6.

[3] S. Michie, L. Atkins, and R. West, *The Behaviour Change Wheel Book - A Guide To Designing Interventions*, 1st ed. Great Britain: Silverback Publishing, 2014. Accessed: Apr. 17, 2022. [Online]. Available: http://www.behaviourchangewheel.com/
