# Supplementary material for: mHealth Physical Activity Intervention for Individuals With Spinal Cord Injury: Planning and Development Processes
Source: JMIR Form Res. 2022 Aug 19;6(8):e34303. doi: 10.2196/34303 (PMC9440410; doi:10.2196/34303)
Supplement: Multimedia Appendix 3 [file formative_v6i8e34303_app3.docx]

**Appendix 3:** How BCTs may target basic psychological needs (BPN) and evidence for use in intervention

| **BCT** | **BPN Target** | **Support** |
| --- | --- | --- |
| Instructions on how to perform behaviour | Competence | Mixed findings when used in combination with other BCTs [1]  Most commonly used among persons with disabilities; difference in effect size [2]  Commonly used to target autonomy [3] |
| Information on antecedents | Competence & Autonomy | Used in SDT studies to target autonomy [3] |
| Information on health consequences | Competence | Effective alone – mixed evidence when used in combination with other BCTs [1]  Commonly used for SDT to target autonomy and competence; effective at improving autonomous motivation [3] |
| Goal setting (behaviour) | Competence & Autonomy | Mixed findings when used in combination with other BCTs [1]  Most commonly used among persons with disabilities [2]  Commonly used to target autonomy in SDT [3] and competence [4] |
| Self-monitoring of behaviour | Competence & Autonomy | Most commonly used among persons with disabilities; showed larger effects than interventions that did not use it [2]  Commonly used in SDT to target competence [3]; targets autonomy and competence [2] |
| Action planning | Competence & Autonomy | Mixed findings when used in combination with other BCTs [1]  Commonly used to target autonomy in SDT [3] and competence [4] |
| Problem solving | Competence & Autonomy | Effective among LTPA interventions for people with SCI (mixed evidence when used in combination with other BCTs) [1]  Most commonly used among persons with disabilities; difference in effect size [2]  Most used in SDT for competence [3], [4] and autonomy [4] |
| Focus on past success | Competence | Used to target competence [4] |
| Verbal persuasion about capability | Competence | Commonly used to increase competence SDT [3], [4] |
| Self-talk | Competence | No information |
| Social support (unspecified) | Relatedness | Mixed findings for LTPA [1]  Social support (practical) – most commonly used among persons with disabilities [2]  Commonly used to increase relatedness [3]; and competence [4] |
| Review goal (behaviour) | Relatedness, Competence | Effective alone and in combination with other BCTs [1]  Commonly used in SDT to target competence [3], [4] |
| Feedback on behaviour | Competence, Relatedness | Larger effects when included than those that did not [2]  Commonly used in SDT to target competence [3], [4] |
| *BCTs Excluded* | | |
| Graded tasks | Competence | Excluded based on feedback from end-users in initial interviews |
| Information about social and environmental consequences | Competence | No improvements with leisure-time physical activity [2] |
| Demonstration of the behaviour | Competence | No improvements with leisure-time physical activity [2] |

**References**

[1] J. R. Tomasone *et al.*, “Physical activity self-management interventions for adults with spinal cord injury: Part 1–A systematic review of the use and effectiveness of behavior change techniques,” *Psychology of Sport and Exercise*, vol. 37, pp. 274–285, 2018, doi: https://doi.org/10.1016/j.psychsport.2018.01.012.

[2] J. K. Ma and K. A. Martin Ginis, “A meta-analysis of physical activity interventions in people with physical disabilities: Content, characteristics, and effects on behaviour,” *Psychology of Sport and Exercise*, vol. 37, pp. 262–273, 2018, doi: https://doi.org/10.1016/j.psychsport.2018.01.006.

[3] F. B. Gillison, P. Rouse, M. Standage, S. J. Sebire, and R. M. Ryan, “A meta-analysis of techniques to promote motivation for health behaviour change from a self-determination theory perspective,” *Health Psychology Review*, vol. 13, no. 1, pp. 110–130, Jan. 2019, doi: 10.1080/17437199.2018.1534071.

[4] S. N. Sweet, M. Rocchi, K. Arbour-Nicitopoulos, D. Kairy, and B. Fillion, “A telerehabilitation approach to anhance quality of life through exercise among adults with paraplegia: Study protocol,” *JMIR Res Protoc 2017;6(10):e202 https://www.researchprotocols.org/2017/10/e202*, vol. 6, no. 10, p. e8047, Oct. 2017, doi: 10.2196/RESPROT.8047.
