# Supplementary material for: mHealth Physical Activity Intervention for Individuals With Spinal Cord Injury: Planning and Development Processes
Source: JMIR Form Res. 2022 Aug 19;6(8):e34303. doi: 10.2196/34303 (PMC9440410; doi:10.2196/34303)
Supplement: Multimedia Appendix 4 [file formative_v6i8e34303_app4.docx]

**Appendix 4:** Outlining how BCTs are incorporated into the intervention content.

| **BCT** | **Intervention Component** | **Rationale** |
| --- | --- | --- |
| Instructions on how to perform behaviour | Quality Participation | Information on quality participation, quality experiences, and factors that contribute to quality participation |
|  | PA Guidelines | Information on PA, types of activities, intensities, guidelines for PA |
|  | Skills | Instruction on how to set up exercise, conduct exercise, videos on how to accomplish resistance training |
| Information on antecedents | Quality Participation | Information on factors that contribute to quality participation |
|  | Coping Planning | Information on barriers that might make PA difficult and how to overcome barriers |
| Information on health consequences | Quality Participation | Information on benefits of quality participation in PA |
|  | Benefits | Information on benefits of PA |
| Goal setting (behaviour) | Goal setting | Information on how to conduct SMART goal setting |
|  | Skills | Attached worksheet on SMART goal setting |
| Self-monitoring of behaviour | Self-monitoring | Information on self-monitoring physical activity |
| Action planning | Action planning | Information on action planning for physical activity |
| Problem solving | Coping planning | Information on how to overcome barriers in physical activity |
|  | Behavioural Support | Information on how to navigate issues in physical activity participation |
| Focus on past success | Beliefs about Capabilities | Focusing on past success to improve confidence in physical activity |
|  | Behavioural Support | Asking participants to reflect on past success after confidence module |
| Verbal persuasion about capability | Behavioural Support | Tell individuals they can do physical activity despite any setbacks (confidence segment) |
| Self-talk | Beliefs about Capabilities | Information on how to change negative self-talk to positive self-talk |
| Social support (unspecified) | Behavioural Support | Encouragement for physical activity, giving information about resources and support in the app |
|  | Peer Support | Encouragement for physical activity, giving information |
| Review goal (behaviour) | Behavioural Support | Discuss goals throughout modules, determine if new goals need to be set |
| Feedback on behaviour | Behavioural Support | Provide feedback on how individual is achieving their goal – affirm and renew confidence throughout modules |
